# SPARQL-enabled identifier conversion with Identifiers.org

**DOI:** 10.1093/bioinformatics/btv064

**Published:** 2015-01-31

**Authors:** Sarala M. Wimalaratne, Jerven Bolleman, Nick Juty, Toshiaki Katayama, Michel Dumontier, Nicole Redaschi, Nicolas Le Novère, Henning Hermjakob, Camille Laibe

**Affiliations:** ^1^European Molecular Biology Laboratory, European Bioinformatics Institute (EMBL-EBI), Wellcome Trust Genome Campus, Hinxton, Cambridge CB10 1SD, UK, ^2^Swiss-Prot group, Swiss Institute of Bioinformatics, Centre Medical Universitaire, 1211 Geneve, Switzerland, ^3^Database Center for Life Science (DCLS), Research Organization of Information and Systems, 178-4-4 Wakashiba, Kashiwa, Chiba 277-0871, Japan, ^4^Stanford Center for Biomedical Informatics Research, Stanford University, CA 94305-5479, USA and ^5^Babraham Institute, Babraham Research Campus, Cambridge, CB22 3AT, UK

## Abstract

**Motivation:** On the semantic web, in life sciences in particular, data is often distributed via multiple resources. Each of these sources is likely to use their own International Resource Identifier for conceptually the same resource or database record. The lack of correspondence between identifiers introduces a barrier when executing federated SPARQL queries across life science data.

**Results:** We introduce a novel SPARQL-based service to enable on-the-fly integration of life science data. This service uses the identifier patterns defined in the Identifiers.org Registry to generate a plurality of identifier variants, which can then be used to match source identifiers with target identifiers. We demonstrate the utility of this identifier integration approach by answering queries across major producers of life science Linked Data.

**Availability and implementation:** The SPARQL-based identifier conversion service is available without restriction at http://identifiers.org/services/sparql.

**Contact:** sarala@ebi.ac.uk

## 1 Introduction

Semantic Web technologies such as the Resource Description Framework (RDF; http://www.w3.org/TR/rdf-primer/) and SPARQL (http://www.w3.org/TR/rdf-sparql-query/) offer a powerful paradigm for publishing and exploring life science data through standardization of format and data access. For example, the open source Bio2RDF (Callahan *et* *al.*, 2013) project converts dozens of public biological databases and datasets from legacy formats into RDF, and provides a mechanism to explore these as Linked Data. Recently, established bioinformatic organizations such as DBCLS (http://togows.dbcls.jp/), NCBI (https://pubchem.ncbi.nlm.nih.gov/rdf/), neXtProt (Chichester *et* *al.*, 2014) and the EMBL-EBI in collaboration with the UniProt consortium (Jupp *et* *al.*, 2014) have made some datasets available in RDF, thereby significantly extending the network of the Linked Open Data.

All efforts use HTTP-based International Resource Identifiers (IRIs) to identify and link data items. This facilitates querying across network-linked resources, but the lack of a universal identifier system requires mappings across all the different identifiers in use. Identifiers.org (Juty *et* *al.*, 2012) provides resolvable persistent IRIs used to identify individual records (based on the existing entity identifiers assigned directly by the data providers). Although some linked data providers such as Bio2RDF and the EBI now make their data available with identifiers.org URIs (or mappings to them), this practice is not widely implemented. Therefore, the identifier mismatch makes it difficult to query multiple datasets simultaneously. String manipulation, supported by SPARQL, may be used for this purpose but requires users to know in advance the IRI types being used in each resource, making it a cumbersome and inefficient solution.

To address the issue of identifier heterogeneity, we have developed a SPARQL-based service that generates on-the-fly identifier mappings for registered IRI patterns. Here, we describe our novel method and demonstrate its functionality through service-enabled federated SPARQL queries. This system offers an automatic way to link and query over a rapidly growing number of semantic web friendly life science datasets.

## 2 Methods

We implemented a SPARQL-based service that generates a set of variant identifiers based on a provided identifier. This service, implemented using the OpenRDF Sesame SPARQL engine (http://www.openrdf.org/), translates an incoming query pattern of the form <subjectIRI>owl:sameAs ?targetIRI and generates a set of triples with the specific subject, predicate, and the generated target IRI. The service queries the curated Identifiers.org Registry to determine the originating data collection, then obtains alternative IRIs patterns, and finally generates and returns alternative IRIs.

## 3 Results

The Identifiers.org Registry contains 531 data collections and over 1300 IRI patterns. The service can be used to find alternative but equivalent IRIs, or check whether two IRIs identify the same concept. For supported data collections, this service eliminates the need to know the set of valid IRI patterns in advance and the need to devise elaborate string manipulation operations in a federated SPARQL query.

The query example below illustrates how the service can be used to query across datasets with different IRI schemes. In this example, we run a federated query to find human proteins from UniProt and their domains from InterPro Bio2RDF that are used in a model’s components (of type SBML species) from BioModels Linked Dataset (Wimalaratne *et* *al.*, 2014). This query can be executed using BioModels SPARQL endpoint (http://www.ebi.ac.uk/rdf/services/biomodels/sparql) and takes around 20 s. The service bridges the gap between the Identifiers.org-specified, Bio2RDF-specified and UniProt-specified identifiers. Further examples are readily available at http://identifiers.org/documentation.

PREFIX rdfs: <http://www.w3.org/2000/01/rdf-schema#>

PREFIX owl: <http://www.w3.org/2002/07/owl#>

PREFIX dcterms: <http://purl.org/dc/terms/>

PREFIX sbmlrdf: <http://identifiers.org/biomodels.vocabulary#>

PREFIX bqbio: <http://biomodels.net/biology-qualifiers#>

PREFIX biomodel: <http://identifiers.org/biomodels.db/>

PREFIX up:<http://purl.uniprot.org/core/>

PREFIX taxon:<http://purl.uniprot.org/taxonomy/>

PREFIX database:<http://purl.uniprot.org/database/>

SELECT DISTINCT ?protein ?protein_domain ?domain_label WHERE {

 

 # query for species annotations in model BIOMD0000000372

 biomodel:BIOMD0000000372 sbmlrdf:species?s.

 ?s sbmlrdf:name?species.

 ?s bqbio:isVersionOf?protein_term.

 

 # query for other IRIs for a given species annotation IRI

 SERVICE <http://identifiers.org/services/sparql>}

  ?protein_term owl:sameAs?protein.

 

 }

 # query for human proteins and their matches to domains

 # in the InterPro database

 SERVICE <http://beta.sparql.uniprot.org/sparql>{

  ?protein a up:Protein;

  up:organism taxon:9606;

  rdfs:seeAlso?protein_domain.

  ?protein_domain up:database database:InterPro.

 }

 

 # query for other IRIs for a given protein domain IRI

 SERVICE <http://identifiers.org/services/sparql>{

  ?protein_domain owl:sameAs?uris.

 

 }

 # query for protein domain labels

 SERVICE<http://interpro.bio2rdf.org/sparql>  {

  ?uris dcterms:title?domain_label.

 }

}

## 4 Discussion

Leveraging the wealth of biomedical big data for discovery requires simple and effective approaches to tame the challenge of working with heterogeneous, overlapping and diverse data. Of particular concern is assignment of different identifiers for identical resources as well as for conceptually identical resources. Identifier integration is the subject of much research that focuses either on integrating conceptually identical objects or their relations (van Iersel *et* *al.*, 2010; Wein *et* *al.*, 2012; Chambers *et* *al.*, 2013). In contrast, our work focuses on the problem of having multiple identifiers for the same database object, which is an emerging issue among semantic web data providers. Our solution is rapid, scalable, and will grow to provide new identifier-based mappings as additional IRI patterns are added to the Identifiers.org Registry.

## 5 Conclusion

This IRI conversion service, provided by Identifiers.org as a SPARQL service, will enable users to focus on asking meaningful questions across biological datasets of interest rather than figuring out how to generate the right identifiers.

## References

[btv064-B1] Callahan A. (2013) Bio2RDF Release 2: improved coverage, interoperability and provenance of life science linked data. Lecture Notes in Computer Science. Vol. 7882, Springer Berlin Heidelberg, pp. 200–212.

[btv064-B2] Chambers J. (2013) UniChem: a unified chemical structure cross-referencing and identifier tracking system. J. Cheminf.*,* 5, 3. 10.1186/1758-2946-5-3 PMC3616875 23317286

[btv064-B3] Chichester C. (2014) Querying neXtProt nanopublications and their value for insights on sequence variants and tissue expression. Web Seman. Sci. Serv. Agents World Wide Web, 29, 3–11.

[btv064-B4] Jupp S. (2014) The EBI RDF platform: linked open data for the life sciences. Bioinformatics*,* 30, 1338–1339. 24413672 10.1093/bioinformatics/btt765 PMC3998127

[btv064-B5] Juty N. (2012) Identifiers.org and MIRIAM Registry: community resources to provide persistent identification. Nucleic Acids Res.*,* 40, D580–D586. 22140103 10.1093/nar/gkr1097 PMC3245029

[btv064-B6] van Iersel M.P. (2010) The BridgeDb framework: standardized access to gene, protein and metabolite identifier mapping services. BMC Bioinformatics*,* 11, 5. 20047655 10.1186/1471-2105-11-5 PMC2824678

[btv064-B7] Wein S.P. (2012) Improvements in the protein identifier cross-reference service. Nucleic Acids Res.*,* 40, W276–W280. 22544604 10.1093/nar/gks338 PMC3394263

[btv064-B8] Wimalaratne S.M. (2014) Biomodels linked dataset. BMC Syst. Biol.*,* 8, 1–9. 25182954 10.1186/s12918-014-0091-5 PMC4423647

